# Editorial: HIV-Associated Immune Activation and Persistent Inflammation

**DOI:** 10.3389/fimmu.2019.02858

**Published:** 2019-12-18

**Authors:** Martin Hoenigl, Harald H. Kessler, Sara Gianella

**Affiliations:** ^1^Division of Infectious Diseases and Global Public Health, University of California, San Diego, San Diego, CA, United States; ^2^Section of Infectious Diseases and Tropical Medicine, Medical University of Graz, Graz, Austria; ^3^Research Unit Molecular Diagnostics, Diagnostic and Research Center for Molecular Biomedicine, Medical University of Graz, Graz, Austria

**Keywords:** non-AIDS events, microbial translocation, fungal translocation, beta-D-glucan, CXCL12, LPS, dysbiosis

While modern antiretroviral therapy (ART) improves health, prolongs survival, and reduces HIV transmission, successfully treated HIV infection remains associated with persistent inflammation and immune dysfunction (1–3). Persistent systemic inflammation is a major contributing factor to HIV-associated morbidity, including neurocognitive impairment (Yu et al.) and non-AIDS events, which result in devastating outcomes and loss of quality of life during aging with HIV infection (4, 5).

The current Research Topic includes in total 11 high-quality manuscripts, ranging from host-response activation during HIV infection (Tjitro et al.), the role of toll-like receptor (TLR)-10 ligand during HIV replication (Henrick et al.), microRNA-19b as a regulator of CD8^+^ T cell functions during HIV infection (Yin et al.), to potentiating the immune response via PD1 checkpoint inhibition (Filaci et al.).

While it is known that HIV infection is characterized by a dramatic depletion of CD4^+^ T cells, a study comparing people with HIV (PWH) with age-matched uninfected controls identified specifically gamma delta (γδ)-T cells as an inflammatory driver in ART-suppressed PWH and provided evidence of distinct “inflamm-aging” processes with and without active HIV replication (Belkina et al.). Depletion of CD4^+^ T cells together with impaired polarization of Th17 cells in the gastrointestinal tract and a massive expansion of activated CD8^+^ T cells causes CD8^+^ T cell-mediated enteropathy (6). In another study of this Research Topic authors also reported dysbiosis and translocation of microbial products in the study participants, which persisted despite long term ART mediated viral suppression (Rhoades et al.). Specifically, the gut microbiome of long term suppressed PWH was enriched in bacterial taxa typically found in the oral cavity suggestive of loss of compartmentalization, while levels of beneficial butyrate producing taxa were reduced. Additionally, prevalence of *Prevotella* negatively correlated with CD4^+^ T-cells numbers, indicating that despite long-term adherence and undetectable viral loads, HIV infection results in significant shifts in gut microbial communities (Rhoades et al.).

Bacteria and fungi are the two most abundant populations of the gut microbiome. While bacterial translocation has been a focus of HIV research for a number of years, fungal translocation recently came into focus as a driver of immune activation, persistent inflammation, and non-AIDS events (7–12). A review included in this supplement evaluated recent literature to untangle the respective roles of circulating lipopolysaccharide (LPS) and (1–>3)-B-D-Glucan (BDG), which are major components of bacterial and fungal cell walls respectively and established biomarker of bacterial and fungal translocation (Ramendra et al.). While LPS is a well-known inducer of innate immune activation, BDG is emerging as a significant source of monocyte and natural killer (NK) cell activation that contributes to immune dysfunction, and both may serve as biomarkers of disease progression and immune activation during ART (Ramendra et al.). Further studies are needed to enhance our understanding of the consequences of elevated LPS and BDG on immune activation, which may inform novel therapeutic strategies against the occurrence of AIDS and non-AIDS events.

Another proposed marker of systemic immune activation during early and chronic HIV infection is plasma CXCL13, which is preferentially secreted by Follicular Helper T cells to attract B cells to germinal centers (Mehraj et al.). A study included in this supplement found that in 114 PWH plasma CXCL13 levels correlated with BDG levels, were higher in those with CMV infection and increased with HIV disease progression (Mehraj et al.). In contrast, early initiation of ART reduced plasma CXCL13 and B cell activation but without normalization. Future studies are needed to compare CXCL13 with more established biomarkers such as soluble urokinase plasminogen activator receptor (suPAR), or sTNFrI, and sTNFrII (2, 4) for prediction of the development of non-AIDS events.

Another study included in this supplement elucidated the role of the immunomodulatory carbohydrate-binding protein Galectin-9 in HIV transcription and in maintaining chronic immune activation during ART-suppressed HIV infection (Colomb et al.). Interactions between cell-surface glycans and glycan-binding proteins (lectins) are key regulators of immunological functions and are involved in several cellular processes. Galectins are a class of lectins that play critical roles in T cell function, and Galectin-9 specifically has recently been recognized to play an essential role in regulating both adaptive and innate defense mechanisms (Colomb et al.). Interestingly, the study found that uncoupling Galectin-9-mediated viral reactivation from undesirable pro-inflammatory effects, using rapamycin, may increase the potential utility of recombinant Galectin-9 within the reversal of HIV latency eradication framework (Colomb et al.).

Finally, this Research Topic includes a paper reporting on a randomized, placebo-controlled, double-blinded trial of the TLR-3 agonist Poly-ICLC in aviremic, ART-treated PWH (Saxena et al.). Poly-ICLC can activate immune cells and induce HIV replication in pre-clinical experiments, but this study for the first time investigated its effect in disrupting HIV latency *in vivo* while simultaneously enhancing innate immune responses. The study found that poly-ICLC was overall safe and well-tolerated (Saxena et al.). Transcriptional analyses revealed upregulation of innate immune pathways in peripheral blood mononuclear cells (PBMCs) following Poly-ICLC treatment, including strong interferon signaling accompanied by transient increases in circulating Interferon gamma-induced protein (IP)-10 (CXCL10) levels (Saxena et al.). These responses generally peaked by 24–48 h after the first injection and returned to baseline by day 8. Overall, CD4^+^ T cell number and phenotype were unchanged, plasma viral control was maintained and no significant effect on HIV reservoirs was observed (Saxena et al.). These finding suggest that Poly-ICLC may be used for inducing transient innate immune responses in treated PWH indicating promise as an adjuvant for HIV therapeutic vaccines (Saxena et al.).

Collectively, the studies described in original research and review articles in this topic describe recent advances and provide optimism for the future of treating HIV-associated inflammation. We hope these articles will stimulate further research with the ultimate goal of improving outcomes for patients with HIV infection.

## Author Contributions

All authors listed have made a substantial, direct and intellectual contribution to the work, and approved it for publication.

### Conflict of Interest

MH received research funding from Gilead that was not related to this Research Topic. The remaining authors declare that the research was conducted in the absence of any commercial or financial relationships that could be construed as a potential conflict of interest.

## References

[1] Deeks SG Verdin E McCune JM. Immunosenescence and HIV. Curr Opin Immunol. (2012) 24:501–6. 10.1016/j.coi.2012.05.004 22658763

[2] Tenorio AR Zheng Y Bosch RJ Krishnan S Rodriguez B Hunt PW Soluble markers of inflammation and coagulation but not T-cell activation predict non-AIDS-defining morbid events during suppressive antiretroviral treatment. J Infect Dis. (2014) 210:1248–59. 10.1093/infdis/jiu254 24795473 PMC4192039

[3] Hoenigl M Chaillon A Little SJ. CD4/CD8 cell ratio in acute HIV Infection and the impact of early antiretroviral therapy. Clin Infect Dis. (2016) 63:425–6. 10.1093/cid/ciw293 27143670 PMC4946020

[4] Hoenigl M Moser C Funderburg N Bosch R Kantor A Zhang Y Soluble Urokinase Plasminogen Activator Receptor (suPAR) is predictive of Non-AIDS Events during Antiretroviral Therapy-mediated Viral Suppression. Clin Infect Dis. (2019) 69:676–86. 10.1093/cid/ciy966 PMC6669298 30418519

[5] Kay A Moser C McKhann A Vargas M Porrachia M Gianella S Soluble suppression of tumorigenicity 2 levels are not predictive of non-AIDS events during antiretroviral therapy-mediated viral suppression. Aids. (2019) 33:1397–9. 10.1097/qad.0000000000002228 31157664 PMC6561120

[6] Arnoczy GS Ferrari G Goonetilleke N Corrah T Li H Kuruc J. Massive CD8 T cell response to primary HIV infection in the setting of severe clinical presentation. AIDS Res Hum Retroviruses. (2012) 28:789–92. 10.1089/AID.2011.0145 22011008 PMC3399555

[7] Hoenigl M Perez-Santiago J Nakazawa M de Oliveira MF Zhang Y Finkelman MA (1 → 3)-β-D-Glucan: a biomarker for microbial translocation in individuals with acute or early HIV infection? Front Immunol. (2016) 7:404 10.3389/fimmu.2016.00404 27752257 PMC5046804

[8] Farhour Z Mehraj V Chen J Ramendra R Lu H Routy JP. Use of (1 → 3)-β-D-glucan for diagnosis and management of invasive mycoses in HIV-infected patients. Mycoses. (2018) 61:718–22. 10.1111/myc.12797 29855088 PMC6175469

[9] Gianella S Letendre SL Iudicello J Franklin D Gaufin T Zhang Y. Plasma (1 → 3)-β-D-glucan and suPAR levels correlate with neurocognitive performance in people living with HIV on antiretroviral therapy: a CHARTER analysis. J Neurovirol. (2019). 10.1007/s13365-019-00775-6. [Epub ahead of print]. 31297727 PMC6923595

[10] Hoenigl M. Fungal translocation: a driving force behind the occurrence of non-AIDS events? Clin Infect Dis. (2019). 10.1093/cid/ciz215. [Epub ahead of print]. 30861074 PMC6938977

[11] Mehraj V Ramendra R Isnard S Dupuy FP Ponte R Chen J. Circulating (1 → 3)-β-D-Glucan is associated with immune activation during HIV infection. Clin Infect Dis. (2019). 10.1093/cid/ciz212. [Epub ahead of print]. 30877304 PMC6938980

[12] Hoenigl M de Oliveira MF Perez-Santiago J Zhang Y Morris S McCutchan AJ. (1 → 3)-β-D-Glucan Levels Correlate With Neurocognitive Functioning in HIV-Infected Persons on suppressive antiretroviral therapy: a cohort study. Medicine. (2016) 95:e3162. 10.1097/MD.0000000000003162 26986173 PMC4839954

